# Evaluating the effect of poly (amidoamine) treated bioactive glass nanoparticle incorporated in universal adhesive on bonding to artificially induced caries affected dentin

**DOI:** 10.1186/s12903-023-03536-4

**Published:** 2023-10-28

**Authors:** Akhil C. Rao, Vijay Venkatesh Kondas, Vidyashree Nandini, Ravi Kirana, Pradeep Kumar Yadalam, Rajalakshmanan Eswaramoorthy

**Affiliations:** 1 Department of Conservative Dentistry and Endodontics, School Of Dental Sciences Krishna Institute, Malkapur, Karad, Maharashtra 415110 India; 2 Department of Conservative Dentistry and Endodontics, SRM Kattankulathur Dental College and Hospital, SRM Institute Of Science And Technology, SRM Nagar, Kattankulathur, Kanchipuram, Chennai, Tamilnadu 603203 India; 3 Department of Prosthodontics and Implantology, SRM Kattankulathur Dental College and Hospital, SRM Institute Of Science And Technology, SRM Nagar, Kattankulathur, Kanchipuram, Chennai, Tamilnadu 603203 India; 4 High Temperature Material Processing Laboratory, Department of Physics and Nanotechnology, SRM Institute of Science and Technology, Kattankulathur, Chengalpattu, Tamil Nadu 603203 India; 5 Department of Periodontics, Saveetha Institute of Medical and Technical Sciences, Saveetha Dental College, Saveetha University, Chennai, 600077 India; 6 Department of Biomaterials, Saveetha Dental College and Hospitals, Saveetha Institute of Medical and Technical Sciences (SIMATS), Chennai, India; 7 Department of Applied Chemistry, School of Applied Natural Science, Adama Science and Technology University (ASTU), PO. 18888, Adama, Ethiopia

**Keywords:** PAMAM dendrimer, Caries-affected dentin, PAMAM-loaded bioactive glass nanoparticles, Universal adhesive, Remineralizing demineralized dentin

## Abstract

**Background:**

The purpose of this study was to evaluate remineralisation and its effect on microtensile bond-strength of artificially induced caries affected dentin (CAD) when treated with a commercial universal adhesive modified with poly(amidoamine) dendrimer (PAMAM) loaded mesoporous bioactive glass nanoparticles (A-PMBG).

**Material and methods:**

Mesoporous bioactive glass nanoparticles (MBG) were synthesised using sol–gel process, where PAMAM was loaded (P-MBG) and added to commercial adhesive at different weight percentages (0.2, 0.5, 1 and 2 wt%). First, rheological properties of commercial and modified adhesives were evaluated. The effect of remineralization/hardness and microtensile bond-strength (MTBs) of those samples that mimicked the rheological properties of commercial adhesives were evaluated using Vickers hardness tester and universal testing machine respectively. Scanning-Electron microscope was used to visualize failed samples of MTBs and remineralization samples. Both evaluations were carried out at 1-,3 and 6-month intervals, samples being stored in stimulated salivary fluid during each time interval.

**Results:**

Addition of nanoparticles altered the rheological properties. With increase in the weight percentage of nanoparticles in commercial adhesive, there was significant increase in degree of conversion, viscosity and sedimentation rate (*p* < 0.05). The 0.2 and 0.5 wgt% groups closely mimicked the properties of commercial adhesive and were evaluated for remineralization and MTBs. After 6 months, 0.2wgt% group showed increased MTBs (*p* < 0.05) and 0.5wgt% group increased remineralization/hardness (*p* < 0.05).

**Conclusion:**

The complex of PAMAM-MBG-Universal adhesive can remineralize the demineralised CAD thereby improving its bond-strength when evaluated for up to 6-months.

## Background

Composites and adhesives are being researched, remodelled and upgraded in terms of material composition and placement techniques [1]. Universal adhesive or multi-mode adhesives, were developed to further simplify adhesive techniques [2]. The acidic functional monomers form stable ionic bonding with calcium present in the hydroxyapatite [3]. Success rates of adhesives and composites depend on the bonding substrate. Higher dentinal organic content makes bonding more challenging compared to enamel.

Caries affected dentin (CAD), a type of reactive dentin substrate most frequently left behind [2]. It develops in response to mild stimuli such as caries and exhibits minor changes in crosslinking of its collagen fibrils [4], decreased microhardness, nano-hardness and modulus of elasticity of matrix with increased water content [5]. Caries depletes calcium from the dentin surface, which is essentially required for nano-layering of MDP-Ca salts [3] which is present in universal adhesives, thus compromising its longevity and durability. Therefore, remineralizing-demineralized CAD would improve adhesive resin bond [6, 7]. One of the most important aspects of treating carious dentin is to ensure that the re-grown mineral is tightly associated with demineralized matrix, restoring dentinal mechanical qualities [6].

Studies have shown that with the bottom-up approach, entire partially demineralized scaffolds including apatite-depleted collagen fibrils can be mineralized [8]. However, it is well established that collagen by itself cannot initiate the biomineralisation process. Two well-known non-collagenous proteins, dentin phosphoprotein and Amelogenin, regulate the natural growth of mineral crystals [9], but do not have the ability to induce remineralisation in mature dentin. Biomimetic analogs that mimic the functions of non-collagenous proteins are required to provide nucleation templates and direct the crystal growth for remineralisation of demineralised dentin [10, 11].

A class of dendrimers called Poly (amidoamine) (PAMAM) are highly branched polymers with numerous terminal reactive groups and have shown to promote dentin remineralisation [12]. They act as a nucleating template and initiate the remineralisation process by binding to dentin collagen and mimicking the functions of organic matrix proteins. They can attract but cannot release calcium and phosphate ions required for initiating the remineralisation process. To facilitate the availability of calcium and phosphate, researchers have tried Amorphous Calcium Phosphate nanoparticles (NACP) with PAMAM dendrimer. However, in these studies, PAMAM was separately applied onto the surface of the dentin to which an adhesive containing NACP was applied [13, 14].

Bioactive glass are being extensively studied for their remineralisation properties [15], by incorporating them in adhesives, composites and GIC [16]. Moreover, porous nature of these nanoparticles facilitate loading of solutions on their surface [17].

In this study, MBG was used as a vehicle to carry PAMAM to inaccessible zones in the demineralised collagen of an artificial caries-affected dentin (A-CAD) substrate. Firstly, change in rheological properties (viscosity, colloidal stability and degree of conversion) of commercial universal-adhesives that could occur on modifying them with different weight percentages (0.2, 0.5, 1 and 2wgt %) of nanoparticles was done. Then by choosing the composition that closely mimicked the rheological property of the commercial adhesive, MTBs and remineralisation effects were evaluated. The null hypothesis of this study would be that, incorporation of poly(amidoamine) coated nanoparticles into the adhesive system did not enhance the remineralising effect and the bond strength.

## Materials and methods

All the experimental protocols were approved by the proceeding of institutional ethics committee, SRM Medical College hospital and Research Centre with ethics clearance number 1797/IEC/2019.

### Synthesis and testing

The MBG was synthesized using sol–gel method following the steps mentioned by Nooney et al. 2004, Yun et al. 2010 [16, 17]. To 1 g of MBG, 20 ml of PAMAM (sigma Aldrich) was added in continuous stirring at slow speed by overnight stirring, as described by bae et al. [18]. The loaded nanoparticles were washed and dried overnight. Samples were characterized before and after addition of PAMAM using XRD, FTIR and TEM. Al the chemicals were purchased from Sigma Aldrich, purity 99%.

A commercially available adhesive (Prime & Bond universal, Dentsply Sirona) with a pH 2.5–3.0 (mild pH adhesive) was chosen for this study. The nanoparticles were added to 10 ml of adhesive at 0.2, 0.5, 1 and 2 wt% [0.2-A-PMBG, 0.5-A-PMBG, 1-A-PMBG, 2-A-PMBG respectively] using a vortex shaker (Tarson 3020 Spinix Vortex Shaker speed 3000 rpm). The obtained adhesives (A-PMBG) were stored in an airtight vial and kept away from light. The samples were tested for their viscosity, degree of conversion, colloidal stability and further analyzed for microtensile bond strength and remineralization.

Viscosity was evaluated using the following method, a set quantity of adhesive was dropped on the glass slab and its diameter was measured. Area obtained was named A. A cover slip was placed over this adhesive drop and a 100 g-weight was applied for 5 min. Diameter after weight placement was measured and area calculated [19]. Delta A corresponds to change in area of the adhesive calculated using the formula:

$$\Delta A = \frac{\left({A}_{after} - {A}_{before}\right)}{{A}_{before}}$$

A smaller ΔA corresponds to a higher viscosity.

Degree of conversion was evaluated by dropping one drop of 10µL adhesive solution on an acetate strip and air-dried for 10 s. FTIR (Shimadzu, IRtracer) of unpolymerized sample was recorded. The adhesive drop was then covered with another acetate strip and cured for 20 s at 1200W/cm^2^. Each specimen was carefully separated from the acetate strips and stored for 24 h. The FTIR of the polymerised specimens were recorded [20, 21]. Degree of conversion was then calculated using the formula:

$$DC\% = \left\{1 - \frac{\left[{C}_{aliphatic} / {C}_{aromatic}\right]}{\left[{U}_{aliphatic} /{U}_{aromatic}\right]}\right\} X 100$$

Where C aliphatic- absorption peak at 1638cm-1 of the polymerized specimen; C aromatic- absorption peak at 1608cm-1 of the polymerized specimen; U aliphatic- absorption peak at 1638 cm-1 of the unpolymerised specimen and; U aromatic- absorption peak at 1608cm-1 of unpolymerised specimen.

Finally, sedimentation behaviour of the adhesive containing nanoparticles with different concentrations was measured using visible light spectrophotometer (labindia UV 3000^+^) at 620nm wavelength. The transmission percentage was recorded every 60min for a minimum of 12h [22].

### Preparation of the tooth samples

The studies were further conducted on the modified adhesives whose properties closely mimic that of the commercial adhesive (0.2wgt% and 0.5wgt%, determined from the part one of the study).

Fifty freshly extracted noncarious human molars were procured from the deposit in the department of oral and maxillofacial surgery. The sample size was calculated using statistical power analysis G*Power 3.1 software and considering ANOVA Repeated measures. The teeth were collected as and when the extraction was performed and no patients were directly involved in the study. The collected teeth were cleaned using an ultrasonic scaler, and stored at 4^O^C in 0.5% chloramine T solution for maximum of 30 days until they could be included in the study. Acrylic bases were made and flat, mid-coronal dentin was exposed using a diamond disk under continuous water flow. Using a diamond abrasive and high-speed hand-piece overall enamel was removed. To obtain a standard smear layer, dentin surfaces were treated using silicon carbide abrasive paper (600 grit) for 60 s. followed by storage of samples in deionized water at 37° C for 24 h. Except for occlusal surface, all other surfaces were covered with two layers of nail varnish.

The specimens were submitted to pH cycling to induce A-CAD. All specimens were individually immersed in the demineralizing solution of pH 4.5 (2.2mM CaCl_2_, 2.2mM NaH_2_PO_4_, 0.05nM acetic acid) for 8 h, 10ml and remineralizing solution of pH 7.0 (1.5mM CaCl_2_, 0.9mM NaH_2_PO_4_, 0.15mM KCL) for 16h, 10ml at room temperature without agitation. The procedure was performed for 14 days and the solution was replaced at each change. Solutions were periodically checked using a pH strip with demineralization with this method reported to be more than 100µm in depth [23, 24].

Twenty-five teeth were assigned to MTBs group to obtain 45 sticks and remaining 25 teeth were allocated to remineralizing group to obtain 45 slabs. An additional 5 rods and 5 slabs were made as replacements in case of any failure occurring during testing.

i. Group A (Control)- Dentin block + unaltered universal adhesive bonding agent. (*n* = 15)
ii. Group B- Dentin block + 0.2-A-PMBG (*n* = 15).
iii. Group C- Dentin block + 0.5-A-PMBG (*n* = 15).

The analysis was carried out at 1,-3 and -6 months intervals, where the samples were stored in stimulated body fluid that contained: 150mmoL/mL CaCl2, 100mmol/mL KH2PO4, 1mol/mL KCl, 100mmol/mL NaN3 and 100mmol/mL HEPES were dissolved in distilled water and pH was adjusted to 7.0 using 100mmol/mL KOH, all the time. It was changed every month and maintained at pH 7.0 [25].

### Microtensile bond strength

Twenty-five teeth samples were restored with universal adhesive and modified adhesives in self-etch mode with no phosphoric acid conditioning as per the manufacturer’s instruction. Following the application of dentin bonding agents, composite (Spectrum Bond, Dentsply Sirona) blocks were prepared on the exposed dentin surface at 1mm/increment to an occlusal height of 4mm and light cured. Samples for microtensile were prepared using hard tissue microtome (Leica SP1600 saw microtome) to obtain 45 sticks of 1 × 1mm dimension. MTBs at 1, -3 and -6 months interval was evaluated using a universal-testing machine (INSTRON ELECTROPLUS E3000) [14].

Fractured samples were checked using SEM (Thermosceintific Apreo S) to evaluate the failure type. Failure types (adhesive, mixed and cohesive) were determined as described by Knobloch et al. Adhesive failure, showing more than 25% of adhesive and/or composite at the dentin side. Mixed failure, showing some areas of Adhesive failure and some areas of cohesive failure. Cohesive failure, showing more than 75% of composite resin/ dentin present on the interface [26].

### Remineralizing effects

Twenty-five teeth were cut approximately 4 × 3 x 2 mm in dimension using a diamond-coated saw under continuous water cooling. A barrier was placed on all surfaces except the one being analysed. The commercial adhesive and modified adhesive were added onto the surface as per manufacturer’s instructions and stored in the simulated body fluid.

The mechanical properties of dentin are by virtue of their mineral content; which means increased mineral content would increase hardness. Hence hardness was measured for three groups at an interval of 1 month, 3 months and 6 months using Vickers diamond Indenter [Shimadzu HMV-G31D, (20 g for 10 s)], making six indents on each sample.

The specimens used to test the hardness were sectioned to evaluate the remineralization. The specimens were fractured to obtain horizontal and vertical sections, which were sputter-coated with gold and examined via SEM (Thermoscientific Apreo S) [25].

Data were entered into Microsoft Excel and analysed using IBM SPSS Statistics for Windows, Version 20 (IBM Corp., Armonk, N.Y., USA). One-way ANOVA and Kruskal Wallis H test followed by multiple comparisons with Tukey’s Honest significant difference and Dunn-Bonferonni post hoc tests were used to analyse continuous and discrete data respectively. The level of statistical significance was determined at *p* ≤ 0.05.

## Results

**Fig. 1:**
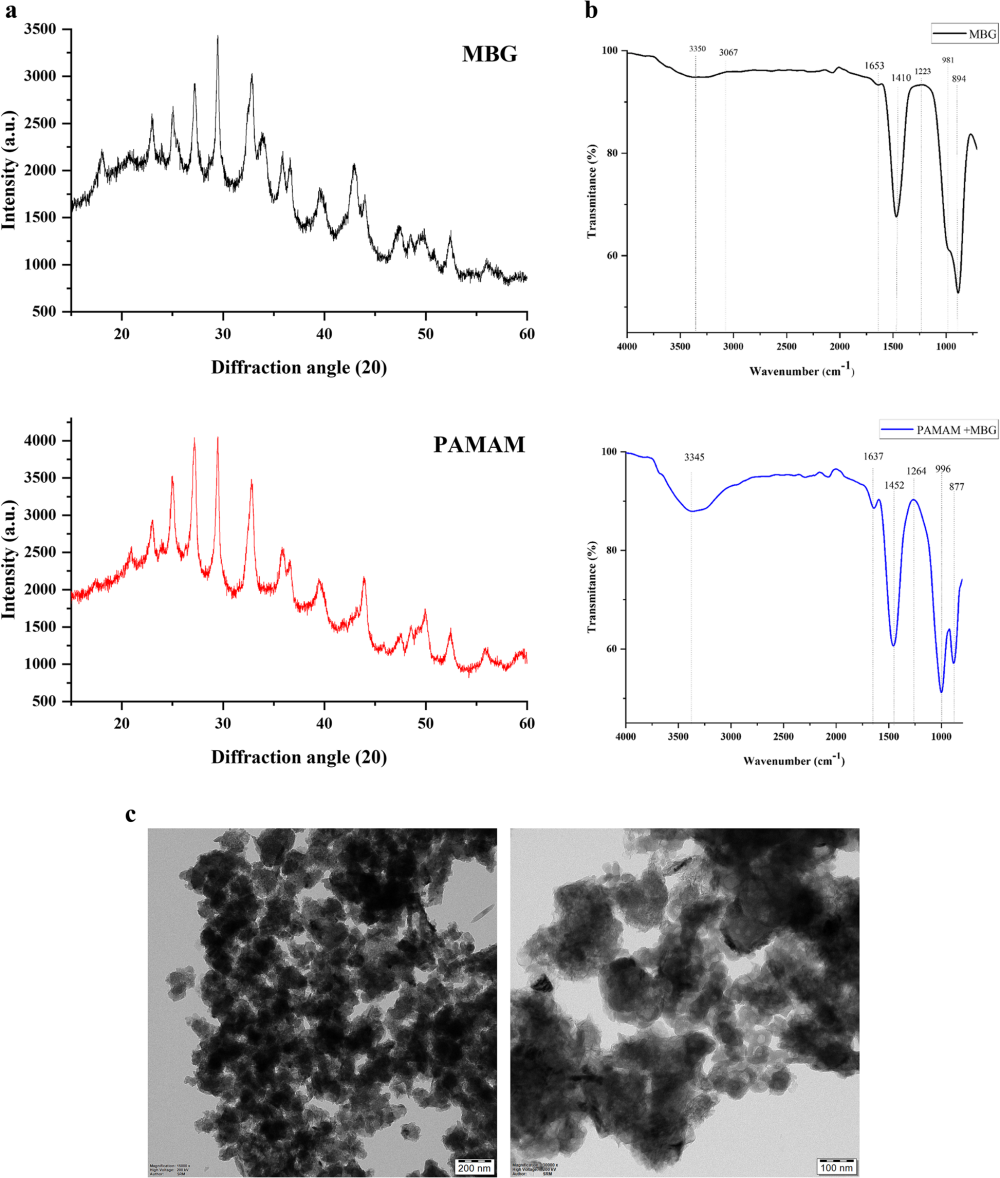
Characterization of MBG and PMBG where; **a**, represents XRD graph; **b**, represents FT-IR graph and **c**, represents Hr-TEM of the samples before and after loading of PAMAM

Microstructural investigation of the nanoparticles and PMBG (Fig. 1).

According to XRD analysis (Fig. 1a) the samples show calcite peak at 29^O^, another peak obtained at 32^O^ is assigned to be apatite according to standard JCPDS cards (#09–0432, #05–0586) [27, 28]. FTIR (Fig. 1b) shows chemical structure of the nanoparticles. The peaks and bands at 1653 cm-1, 1223 and 894 cm-1 are associated with Si–O-Si vibration. Band at 981 cm-1 were due to Si–OH bending deformation and stretch at 1410 cm-1 depict carbonate groups present in the precursor. The peak at 3350 cm-1 was attributed to OH group vibration. The FTIR spectra of PAMAM loaded MBG is similar to that of MBG. Stretching bond of C-H represented by peak 3067 and CH2 vibrations were represented by peaks 2940 and 1465. Whereas, peak at 996 is related to phosphate group due to P-O absorption [17, 27]. TEM analysis (Fig. 1c) confirmed the diameter of nanoparticles ranged from 35 nm – 60 nm, with an ordered mesoporous structure. The particle size was relatively homogeneous. In PAMAM loaded MBG, MBG molecules were coated with PAMAM [18].

### Effects on rheological properties of commercial adhesive

**Fig. 2:**
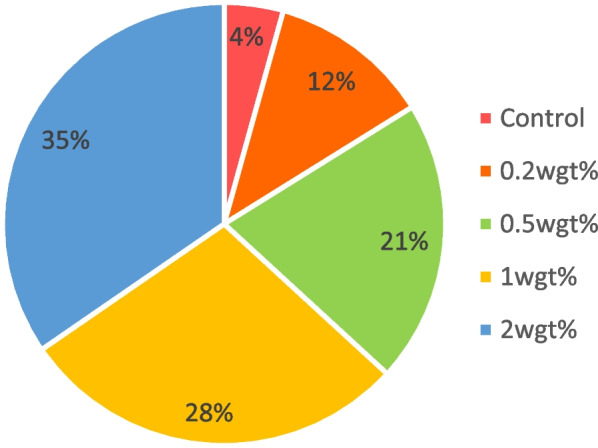
Pie chart depicting the viscosities of control and experimental groups. Where the viscosity of 0.2wgt% group was comparable to that of control. Viscosity seemed to have decreased when the concentration of nanoparticles are increased (*p* < 0.05)

**Fig. 3:**
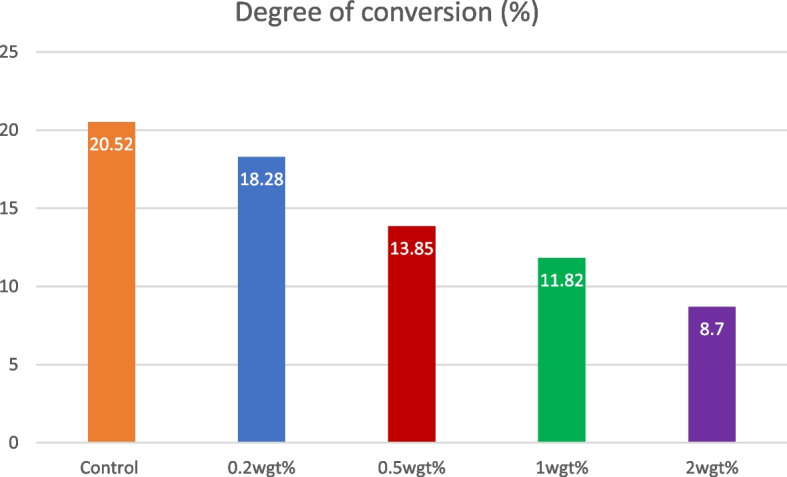
Is a bar diagram that depicts the degree of conversion between each group, where degree of conversion of control group was comparable to that of 0.2 and 0.5wgt% groups

Data obtained for the viscosity and degree of conversion were statistically significant between control and experimental groups (*p* < 0.05). The viscosity of the control group was 6.40 $\pm$ 0.31mm^2^ which was closely related 0.2-A-PMBG (5.56 $\pm$ 0.38) (*p* < 0.05). The weight percentage of the nanoparticle added is inversely proportional to that of viscosity of the adhesive resin (Fig. 2). The degree of conversion for control (23%) was comparable to that of 0.2-A-PMBG (18%). Percent degree of conversion of the adhesive resin decreased as the weight of nanoparticles added to the adhesive resin increased in the order 2-A-PMBG < 1-A-PMBG < 0.5-A-PMBG i.e., 3%, 8% and 18% respectively (*p* < 0.05) (Fig. 3).

**Fig. 4:**
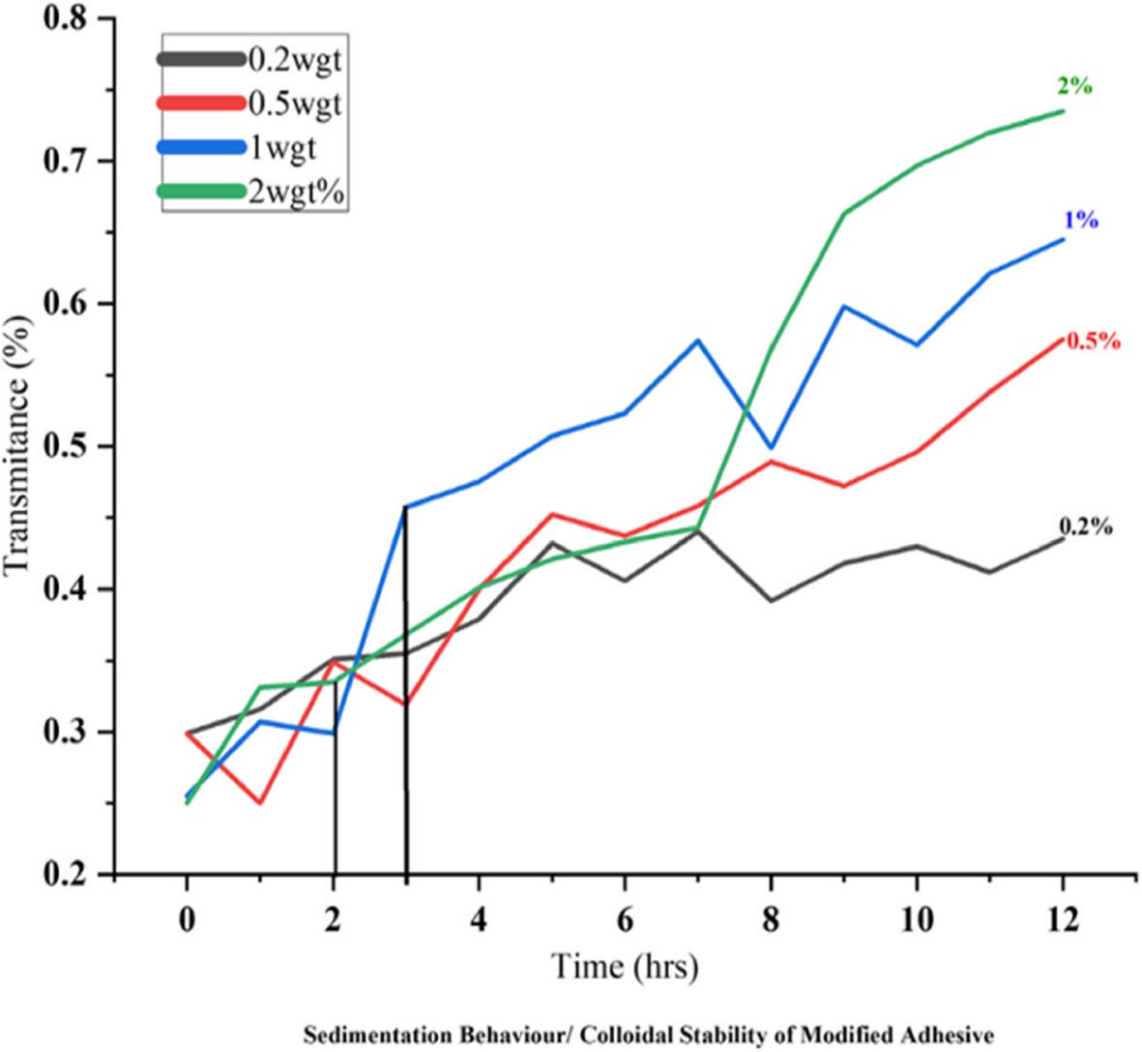
Shows transmittance percentage for different experimental groups. Transmittance percentage increased at 2 h and 3 h for 2wgt% and 1wgt% respectively, indicating the sedimentation of nanoparticles by 2–3 h for these group. No sedimentation was seen for 0.2wgt% at the end of 12 h

The sedimentation behaviour, measured by spectrophotometer, of nanoparticle incorporated in experimental adhesive at different concentrations is shown in Fig. 4. There was increased sedimentation of the particles with time in the adhesive solution. Although there was gradual sedimentation of the nanoparticles seen with 0.2-A-PMBG, the transmission during 12 h did not reach maximum. However, sedimentation rate of nanoparticles dramatically increased nearly after 2-3 h for 1-A-PMBG and 2-A-PMBG and 6 h for 0.5-A-PMBG.

### Microtensile Bond Strength:

Around 4 bonded sticks were obtained per tooth, two (1 × 1 mm, 8 mm length) of the longest sticks were selected for MTBs analysis. At 6 months interval, the mean bond strength for control, 0.2-A-PMBG and 0.5-A-PMBG were 15.82 $\pm$ 1.91MPa, 24.29 $\pm$ 2.90MPa and 19.46 $\pm$ 1.62MPa respectively. Statistically significant results were seen at 6 months interval (*p* < 0.05).

**Fig. 5:**
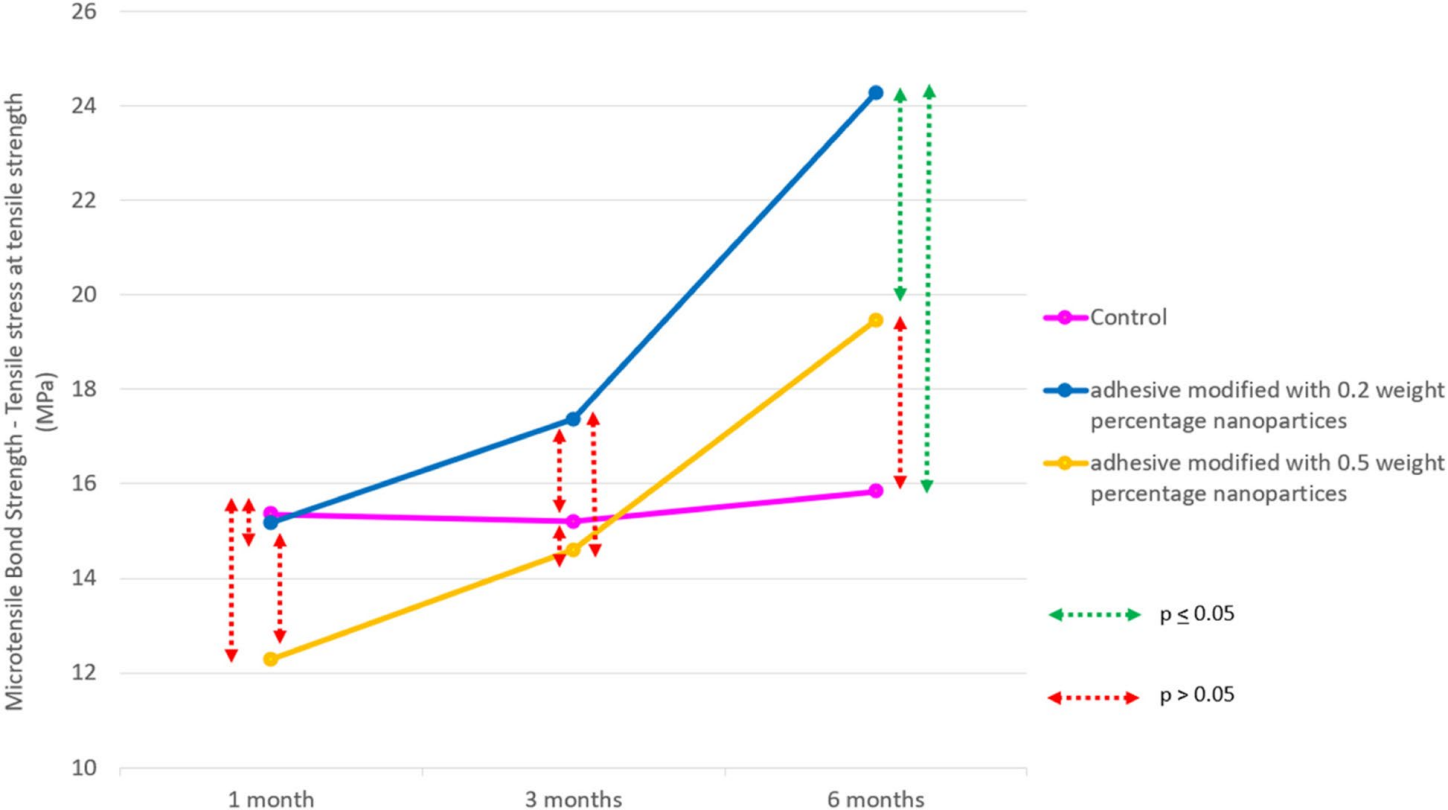
Represents line diagram depicting microtensile bond-strength of the samples

**Fig. 6:**
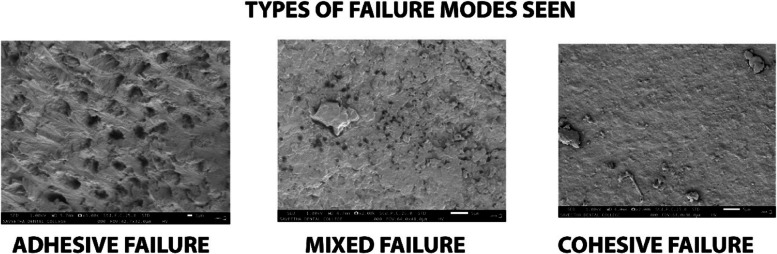
Shows SEM analysis of mode of failures

Intergroup comparison showed gradual increase in the bond-strength values for both the experimental groups (0.2-A-PMBG: 15.18 $\pm$ 2.11MPa, 17.38 $\pm$ 2.15MPa and 24.29 $\pm$ 2.90MPa; 0.5-A-PMBG: 12.82 $\pm$ 1.86MPa, 14.60 $\pm$ 1.66MPa and 19.46 $\pm$ 1.62MPa) at 1-,3 and 6-months interval (Fig. 5). Adhesive failures were the most common failure at all the time periods. Two cohesive failure and four mixed failures were seen in total at the end of 6-months (Fig. 6).

### Microhardness and remineralization effect

**Fig. 7:**
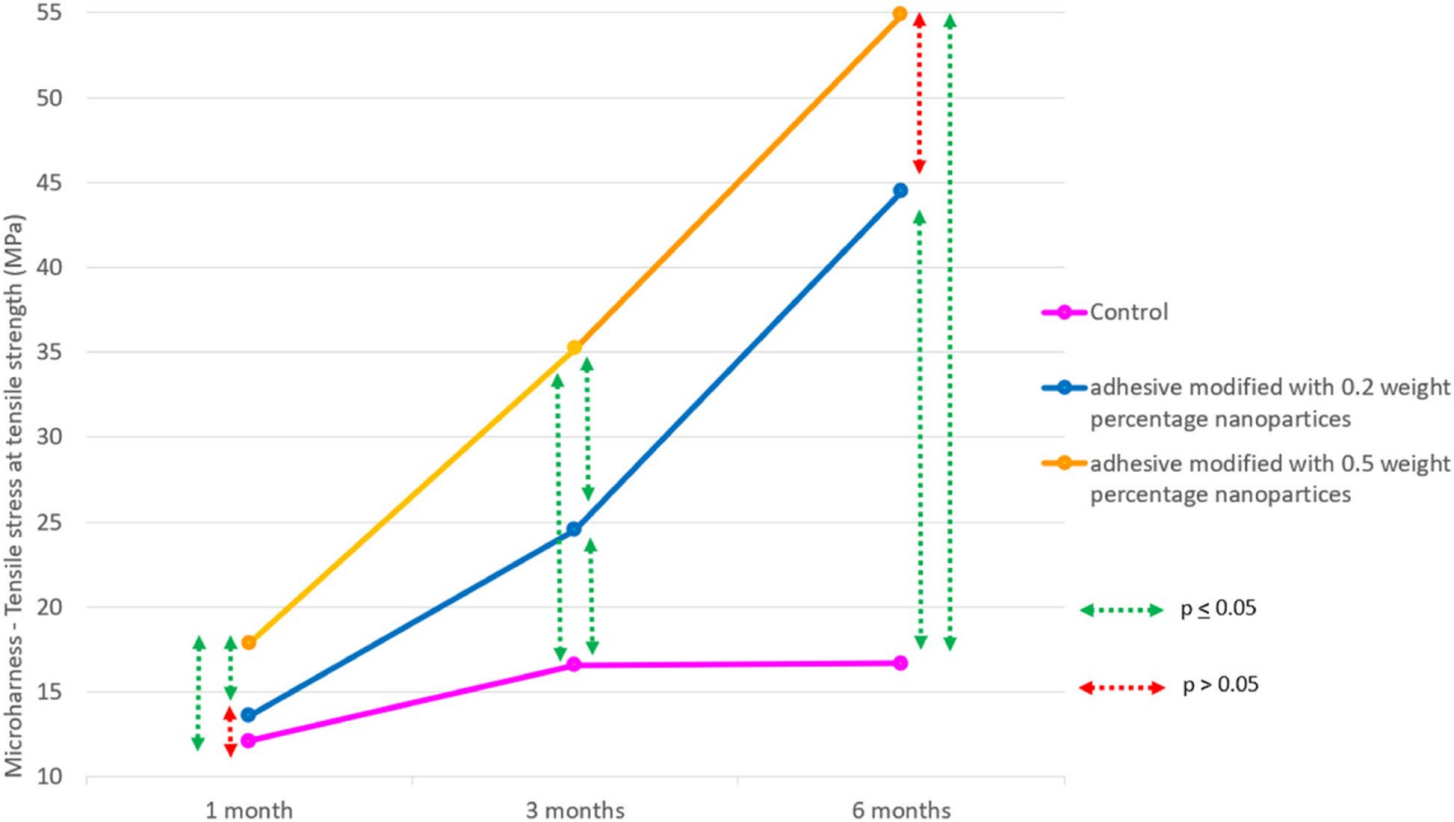
Shows line diagram depicting microhardness of the samples

**Fig. 8:**
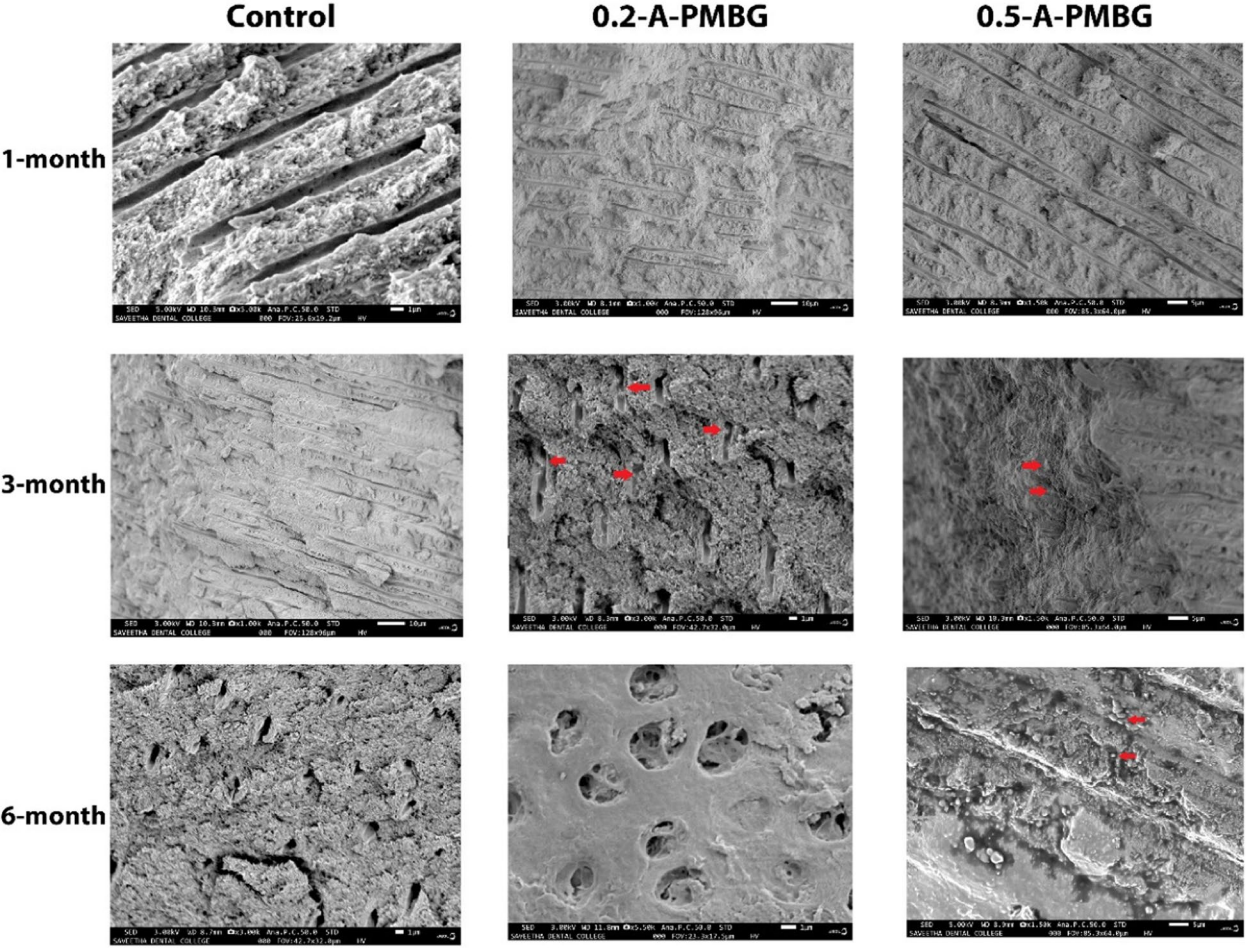
Scanning electron microscopy images of dentine surfaces at 3 different intervals. At one month interval, intertubular and peritubular dentine along with normal tubular pattern is seen in the fractured dentine samples. The red arrows, at 3-month interval shows the pattern of mineral deposition, and at 6-month interval, the red arrow indicates large spherical mineral aggregates deposited on the surfaces of the dentinal tubules, with almost to complete obliteration of tubular openings

Inter-group comparison revealed steady increase in hardness values for experimental groups when compared to the control at the end of 6 months, with 0.5-A-PMBG showing highest hardness values at the end of 6-months (*p* < 0.05) (Fig. 7). The dentin slabs that showed the maximum hardness were selected for SEM analysis to evaluate the remineralisation. In 30 days’, no minerals deposits were seen in the dentinal tubules for all the 3 groups. At 3-month interval, deposition of minerals was seen on the opening of the dentinal tubules in the experimental groups. No mineral depositions were seen in the control group. At 6-month interval, well defined large spherical mineral crystals were seen in 0.5-A-PMBG and 0.2-A-PMBG. Images of mineral deposited at 1, 3 and 6-month interval is shown in Fig. 8.

## Discussion

One of the main issues with caries affected dentin is its substrate composition that potentially compromises the longevity and durability [5] of the adhesive bond. Remineralizing the demineralised caries affected dentin could potentially improve the bond strength [6, 7]. Based on the observations made from this study, the null hypothesis was rejected, as PAMAM-loaded Bioactive glass nanoparticles could successfully increase the remineralisation and micro-tensile bond-strength of CAD over a period of 6-months.

Primarily, stability of the commercial adhesive when different weight percentages of nanoparticle added was evaluated. The modified adhesive seemed to become viscous as the percentage of loaded nanoparticles increased in the commercial adhesive. 1-A-PMBG and 2-A-PMBG showed higher viscosity, higher degree of conversion and lower colloidal stability when compared to unaltered commercial adhesive. Alteration in rheological properties as reported by Shortall et al. [29] is attributed to increased contact angle and difference in refractive index of filler and resin matrix. This could explain the increased viscosity and degree of conversion when the percentage of nanoparticles added increased.

MTBs of experimental group was much higher than the control. Dental adhesives are resin monomer solutions that allow the resin to interact with the dental substrate [30]. The primary goal of any bonding strategy is to achieve a close link between the adhesive systems and teeth [31]. For predictable bonding to occur, the liquid adhesive must adequately wet the adherent [32]. The rheological properties of dental adhesives have significant effect on the bond-strength of the adhesive. Hass et al. [33], demonstrated that degree of conversion inside the hybrid layer was found to be correlated MTBs. Similar observations were made in terms of viscosity of the adhesive resin and formation of hybrid layer [34]. This would explain why the 0.5-A-PMBG showed lower MTBs and a higher hardness value when compared to 0.2-A-PMBG.

In biomimetic remineralization, remineralization of demineralised dentin collagen is induced by liquid-like nanoprecursors particles that are stabilised by biomimetic analogs of non-collagenous proteins [35]. Hence, this nucleation can aid deposition of minerals in demineralized dentin, in a structured fashion, making the substrate desirable for bonding. Result of this study indicates remineralization could increase the micro-tensile bond-strength of A-CAD. Which can be corroborated with previous studies conducted with different remineralizing agents applied separately on the tooth structure before application of the bonding agent [36, 37]. Previous studies used Amorphous calcium phosphates (CPP ACP), fluoride and calcium-phosphate derivatives. In the following study, MBG were used as a remineralizing agent.

Along with its ability to remineralize, due to its morphology, MBG act as an excellent delivery system. Incorporation of PMBG into a commercial adhesive system could facilitate carrying of PAMAM polymer through the gap zone [38] in the collagen where PAMAM could bind to the collagen fibrils and MBG provide calcium and phosphate ions to initiate the remineralization process, which was corroborated with result of this study.

The monomers used in the universal adhesives have a tendency to link ionically to calcium-salts present in the hydroxyapatite, leading to formation of stable salt (10-MDP-Calcium salt). Formation of this stable salt strengthens the interface, thereby enhancing the bond stability. In addition to the acidic functional monomer, these adhesives are supplied in four different pH levels. The adhesive belligerence, mostly its pH, determines its interaction with the smear layer and to what extent the dentin substrate demineralizes [39–41]. Prime and bond universal, used in this study, is a mild universal adhesive with a pH≈2 that facilitates chemical bonding of the functional group and available hydroxyapatite in dentin collagen [42, 43].

Joves et al., [44] compared an A-CAD model to natural CAD of human teeth for in-vitro bonding tests. They concluded that despite their dissimilar morphologies, A-CAD model and natural CAD had equal mineral density and MTBs. The method to induce artificial caries by pH-cycling has been discussed in several studies, where A-CAD substrate had similar morphology to that of natural CAD substrate. In addition to this, Joves et al., found that type of caries had no effect on MTBs. These data support the use of pH-cycling as suitable method to induce A-CAD [23, 24, 45].

Transmission electron microscopy analysis (TEM), has shown to be more accurate in determining the intrafibrillar remineralization, however, data obtained from micro-hardness tests and SEM analysis did confirm remineralization of the dentin substrate. Though the pH cycling model reliably induces artificial caries, it does not simulate the exact physiologic changes that occur in clinical situations. Responses of pulp-dentin complex in formation of sclerotic dentin, tertiary dentin and action of MMPs in response to dentin conditioning could affect the adhesion stability, making it another limitation of this study [46] In this study, PAMAM loaded nanoparticles were stable in a solution at 0.2wgt%, long term stability of the solution needs to be evaluated.

Results of this study confirm the benefits of modifying a commercial adhesive with 0.2wgt% PMBG in increasing the bond-strength to A-CAD. It was also observed that with increased concentrations of PMBG, the samples showed increased remineralization effect. Increasing the weight percentage of nanoparticles in commercial adhesives could be challenging as they are marketed in their most stable from, however, incorporation of these modified nanoparticles at manufacturing level could enable higher filler loading, which could improve bond-strength.

## Conclusion

In this investigation, the rheological properties were not significantly changed by 0.2-A-PMBG and 0.5-A-PMBG; however, there were negative effects from increasing the weight percentage of nanoparticles. MTBs and remineralization significantly increased in 0.2-A-PMBG and 0.5-A-PMBG. However, when evaluated over a 6-month period, there was a drop in the MTBs and an increase in remineralization of the samples with increasing weight percentage of the nanoparticles. As a result, within the constraints of this investigation, nanoparticles in the adequate concentration demonstrated promising results in boosting MTBs and remineralization in simulated CAD without modifying the rheological properties of the universal adhesive.

## Data Availability

The datasets used and/or analyzed during the current study available from the corresponding author on reasonable request. All data underlying the findings and outcome are presented as part of the article and no supplementary source data are required.

## Abbreviations

PAMAM: Poly(amidoamine) dendrimer
MBG: Mesoporous bioactive glass nanoparticles
A-PMBG: Commercial adhesive modified with PAMAM-loaded MBG
MTBS: Microtensile bond-strength
CAD: Caries-affected dentin
A-CAD: Artificially-induced CAD
